# Effects of Ribosomal Protein S10 Flexible Loop Mutations on Tetracycline and Tigecycline Susceptibility of *Escherichia coli*

**DOI:** 10.3389/fmicb.2021.663835

**Published:** 2021-06-18

**Authors:** Norbert Izghirean, Claudia Waidacher, Clemens Kittinger, Miriam Chyba, Günther Koraimann, Brigitte Pertschy, Gernot Zarfel

**Affiliations:** ^1^Diagnostic and Research Center for Molecular BioMedicine, Medical University of Graz, Graz, Austria; ^2^Institute of Molecular Biosciences, University of Graz, Graz, Austria; ^3^Biotech Campus Tulln, University of Applied Sciences Wiener Neustadt, Tulln, Austria; ^4^BioTechMed-Graz, Graz, Austria

**Keywords:** 16S rRNA, helix 31, tigecycline, tetracycline, ribosomal protein S10, rpsJ

## Abstract

Tigecycline is a tetracycline derivative that is being used as an antibiotic of last resort. Both tigecycline and tetracycline bind to the small (30S) ribosomal subunit and inhibit translation. Target mutations leading to resistance to these antibiotics have been identified both in the 16S ribosomal RNA and in ribosomal proteins S3 and S10 (encoded by the *rpsJ* gene). Several different mutations in the S10 flexible loop tip residue valine 57 (V57) have been observed in tigecycline-resistant *Escherichia coli* isolates. However, the role of these mutations in *E. coli* has not yet been characterized in a defined genetic background. In this study, we chromosomally integrated 10 different *rpsJ* mutations into *E. coli*, resulting in different exchanges or a deletion of S10 V57, and investigated the effects of the mutations on growth and tigecycline/tetracycline resistance. While one exchange, V57K, decreased the minimal inhibitory concentration (MIC) (Etest) to tetracycline to 0.75 μg/ml (compared to 2 μg/ml in the parent strain) and hence resulted in hypersensitivity to tetracycline, most exchanges, including the ones reported previously in resistant isolates (V57L, V57D, and V57I) resulted in slightly increased MICs to tigecycline and tetracycline. The strongest increase was observed for the V57L mutant, with a MIC (Etest) to tigecycline of 0.5 μg/ml (compared to 0.125 μg/ml in the parent strain) and a MIC to tetracycline of 4.0 μg/ml. Nevertheless, none of these exchanges increased the MIC to the extent observed in previously described clinical tigecycline-resistant isolates. We conclude that, next to S10 mutations, additional mutations are necessary in order to reach high-level tigecycline resistance in *E. coli*. In addition, our data reveal that mutants carrying S10 V57 exchanges or deletion display growth defects and, in most cases, also thermosensitivity. The defects are particularly strong in the V57 deletion mutant, which is additionally cold-sensitive. We hypothesize that the S10 loop tip residue is critical for the correct functioning of S10. Both the S10 flexible loop and tigecycline are in contact with helix h31 of the 16S rRNA. We speculate that exchanges or deletion of V57 alter the positioning of h31, thereby influencing both tigecycline binding and S10 function.

## Introduction

Tigecycline is an antibiotic of last resort. It is, beside colistin, far too often the last antibiotic that is effective, especially in infections with carbapenem-resistant Enterobacteriaceae (with some exceptions like *Proteus* spp., which is naturally resistant to tigecycline) (Gales and Jones, 2000; Meagher et al., 2005).

Tigecycline is the first member of the glycylcycline class, which is derived from tetracycline, more precisely from the semi-synthetic tetracycline minocycline. Alike tetracycline, tigecycline binds to the 30S ribosomal subunit, albeit with higher affinity, and inhibits translation elongation by preventing delivery of tRNA to the ribosomal A site (Bergeron et al., 1996).

**FIGURE 1:**
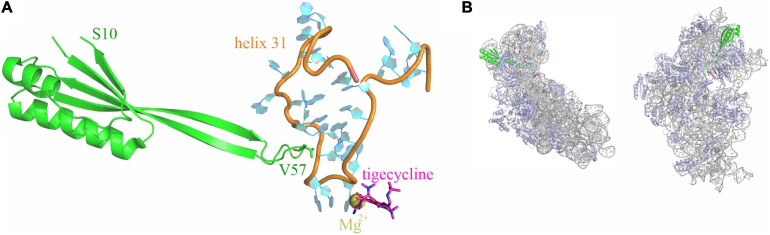
Tigecycline and ribosomal protein S10 bind to helix h31 of the 16S rRNA. **(A)** Relative positioning of ribosomal protein S10 (green), helix 31 (orange and cyan), and tigecycline (magenta; coordinates a Mg^2+^ ion) within the X-ray structure of a tigecycline-bound *E. coli* 30S subunit [PDB 5j91 (Cocozaki et al., 2016)]. The S10 loop tip residue V57 is indicated. **(B)** The same elements as in A are shown in the context of the entire 30S subunit. 16S rRNA is displayed in gray color, ribosomal proteins other than S10 are shown in blue. The left display is in the same orientation as in (A), the right display is rotated into the solvent-exposed side display.

Structures of tigecycline bound to the 70S ribosome showed that the binding site of tigecycline comprises nucleotides of helices h31 and h34 of the 16S rRNA located at the base of the head domain of the 30S ribosomal subunit (Figure 1; Bergeron et al., 1996; Gales and Jones, 2000; Bauer et al., 2004; Jenner et al., 2013).

The higher affinity of tigecycline compared to tetracyline results in a more efficient block of bacterial protein synthesis. Even more importantly, most tetracycline resistance mechanisms, like *via* ribosomal protection proteins (e.g., TetM in *Escherichia coli*) or specific tetracycline efflux pumps (e.g., TetL), are not or at least less effective against tigecycline (Jenner et al., 2013; Fiedler et al., 2016). Most documented mutations leading to tigecycline resistance are chromosomally encoded, like mutations in genes regulating efflux pumps (e.g., AcrAB-TolC or CusR). There are also rare cases in which tigecycline resistance occurs on mobile elements, as reported for efflux pumps, as well as for a TetX variant, an enzyme that normally breaks down tetracyclines (Fiedler et al., 2016; Liu et al., 2020). Tigecycline-resistant mutations have also been observed in RNA or protein components of the 30S ribosomal subunit (Yuhan et al., 2016; Argudin et al., 2017; Chen et al., 2020).

Tigecycline-resistant 16S rRNA mutations lie in helices h31 and h34 in proximity to the tigecycline binding site. Remarkably, G966, the residue in h31 that is in direct contact with tigecycline, is subject to methylation by the methyltransferase RsmD, and mutations in this enzyme have been found to cause resistance to tigecycline in *Streptococcus pneumoniae* (Lupien et al., 2015).

Notably, also mutations in genes *rpsC* and *rpsJ*, encoding ribosomal proteins S3 and S10, lead to tigecycline resistance. Neither of these ribosomal proteins is in direct contact with tigecycline; however, S3 and S10 interact with 16S rRNA helices h34 and h31, respectively. Reports of *rpsJ* mutation-mediated tigecycline resistance include both clinical isolates and mutants generated under laboratory conditions upon tigecycline pressure. All reported mutations occur in (or include) an unstructured loop of S10, which is in direct contact with h31 (Figure 1; Beabout et al., 2015; Lupien et al., 2015; Fang et al., 2016; He et al., 2018).

**FIGURE 2:**
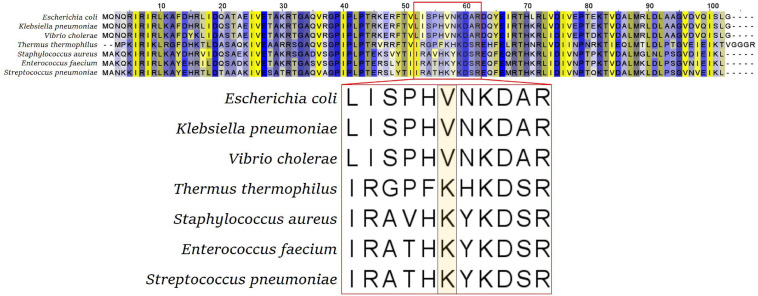
S10 alignment of selected bacterial species. The flexible loop sequence is indicated by the red box, with the tip amino acid (valine V or lysine K) highlighted in the enlarged representation (bottom panel).

Although this flexible loop is present in all kingdoms of life, its sequence is not very conserved. The tip amino acid of this flexible loop is a valine (V) in most gram-negative bacteria, including Enterobacteriaceae, Pseudomonadales, Vibrionales, Neisseriales, and Bacteroidales, while it is a lysine (K) in gram-positive bacteria and in *Thermus thermophilus* (Figure 2). Isolated tetracycline and tigecycline-resistant mutants showed a variety of amino acids to which this tip residue was exchanged. In Enterobacteriaceae, tigecycline-resistant *rpsJ* variants with exchanges of the tip valine residue (V57) to either aspartic acid (D), leucine (L), or isoleucine (I) were found (Villa et al., 2014; Beabout et al., 2015; Fang et al., 2016; Li et al., 2016). V57L is also reported from doxycycline-resistant *Vibrio cholerae*; in that study, tigecycline was not tested (Narendrakumar et al., 2020). In gram-positive cocci, there are reports of exchanges of K57 to arginine, methionine, glutamine, glutamic acid, and asparagine connected with tigecycline resistance. Also, deletions of parts of the loop and mutation of other residues in the tip of the loop were reported for *Staphylococcus*, *Streptococcus*, and *Enterococcus*, in particular mutations of Y58 and K60 (Beabout et al., 2015; Cattoir et al., 2015; Argudin et al., 2017; Haim et al., 2017).

Most of these mutants were generated by natural or artificial selection; therefore, mutations in additional genes might be present that contribute to the resistance of these strains. Indeed, there are reports of tigecycline-resistant clinical isolates carrying, beside S10 mutations, also mutations in additional tetracycline/tigecycline resistance genes, like *tetM* (Cattoir et al., 2015). To exclude such additional influences due to mutations in genes other than *rpsJ*, previous studies aimed at integrating *rpsJ*-mutant alleles into the chromosome of genetically defined laboratory strains. These efforts were successful for *Enterococcus* and *Neisseria*, confirming the effects of S10 loop mutations on the susceptibility to tigecycline or tetracycline in these bacteria, while previous studies failed to integrate *rpsJ* mutant alleles into the chromosome of *E. coli* strains (Hu et al., 2005; Beabout et al., 2015).

In this study, we developed a strategy for the chromosomal integration of *rpsJ* mutant alleles into the *rpsJ* operon. Using this strategy, we created a set of different mutants of an *E. coli* laboratory strain in which V57 of S10 was exchanged. Surprisingly, the exchange of V57 to lysine, the amino acid present in gram-positive organisms and *Thermo thermophilus* at this position, led to a decreased minimal inhibitory concentration (MIC) and hence hypersensitivity to tetracyclines. All but one of the other exchanges, including the ones reported previously in resistant isolates, resulted in an increased MIC to tigecycline, minocycline, and tetracycline. However, in no case was the MIC increase high enough to classify the mutant as resistant according to EUCAST criteria. We conclude that, next to S10 mutations, additional mutations are necessary to reach high-level tigecycline resistance in *E. coli*. Moreover, all S10 mutants showed growth defects and, in most cases, also thermosensitivity. We speculate that in addition to the role of the amino acid in position 57 of S10 in determining the extent of tigecycline susceptibility, it is also important for the correct functioning of S10.

## Materials and Methods

### Construction of *rpsJ* Mutants

#### Strains and Plasmids

The *E. coli* laboratory strain used in this study was BW 25113. To increase the rate of homologous recombination, the recombination helper plasmid pKD46 was used. pKD46 carries a temperature-sensitive replicon (*rep*A101ts), an ampicillin resistance gene, and lambda Red genes (*exo, bet*, and *gam*) for homologous recombination under the control of an arabinose-inducible promoter. Plasmid pET-28 a (+), containing a kanamycin resistance marker, was used as template for PCR amplification of the kanamycin resistance gene. Strains *E. coli* BW P3 and *E. coli* BW P6, carrying an S10 V57L and an S10 V57D exchange, respectively, were provided by Yousif Shamoo. Both strains were generated by selection of *E. coli* BW 25113 under tigecycline pressure (Beabout et al., 2015).

#### Integration of the Kanamycin Resistance Gene Into the *rpsJ* Operon

**FIGURE 3:**
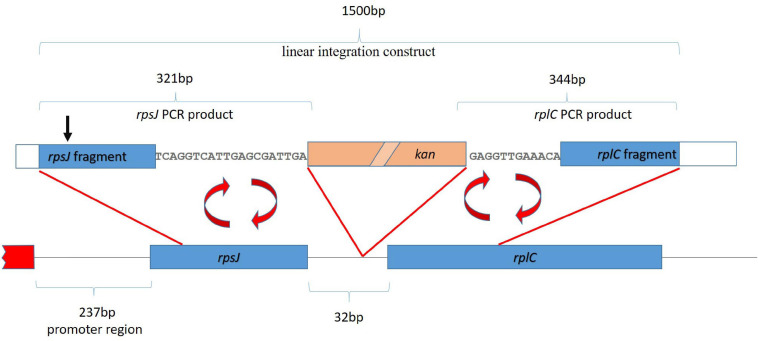
Strategy for homologous recombination and introduction of a kanamycin resistance gene (kan, salmon colored box). The first ∼2 kb of the *rpsJ* operon, including the first two genes, *rpsJ* and *rplC*, are depicted. The red box depicts a region outside the operon. The kanamycin resistance gene was inserted into the non-coding region between *rpsJ* and *rpsC*. For the integration by homologous recombination, a 1,500-base-pair-long linear fragment, indicated on top of the figure, was generated by fusion of the indicated *rpsJ* and *rpsC* PCR fragments with the kanamycin resistance gene. The position corresponding to S10 V57 is indicated (black arrow).

A cassette suitable for integration of the kanamycin resistance gene into the *rpsJ* operon, 20 bp after the *rpsJ* and 11 bp before the *rplC* gene, was constructed (see also Figure 3). To this end, a linear fragment containing the kanamycin resistance gene flanked by *loxP* sites, followed by DNA sequences homologous to the region before and after the site of planned recombination into the *rpsJ* operon was generated *via* a multistep PCR procedure using the primers listed in Supplementary Table 1. First, three separate fragments were amplified. (1) A fragment homologous to the last 282 bp of the *rpsJ* reading frame, followed by 20 bp of the *rpsJ*-*rplC* intergenic region (302 bp together), was amplified from the *E. coli* strain BW 25113. A 19-bp overlap with the *loxP* site of the kanamycin resistance cassette was introduced *via* the reverse primer (resulting in a 321-bp PCR product, subsequently referred to as “*rpsJ* PCR product” for simplicity reasons). (2) A fragment homologous to the 12 bp of the intergenic region before *rplC*, followed by 288 bp of the *rplC* reading frame (300 bp together), was amplified and a 44-bp overlap with the kanamycin resistance cassette and *loxP* site was introduced *via* the forward primer (resulting in a 344-bp PCR product subsequently termed “*rplC* PCR product”). (3) The kanamycin resistance gene was amplified using the pET-28 a plasmid as template, and flanking *loxP* sites were introduced *via* the primers.

Twenty-five-microliter PCR reactions were assembled containing 10 μl of Q5 5× reaction buffer (NEB), 2.0 μl of dNTP-mix (2 mM), 0.1 μl of Q5 polymerase (NEB), 2.5 μl of template DNA (15–20 ng), 1 μl of forward and 1 μl of reverse primer (10 pmol/μl each), and 8.4 μl of water. The program started with an initial denaturation for 5 min at 98°C, followed by 35 cycles of denaturation at 98°C for 20 s, annealing at 52°C for 45 s, and extension at 72°C for 60 s, followed by a 10-min 72°C final extension step and cooling to 4°C. PCR products were then separated on an 1% agarose gel for 1 h at 85 V, excised from the gel and purified with Wizard^®^ SV Gel and PCR Clean-Up System (Promega, Walldorf, Germany).

In the second step, equal amounts of the purified overlapping *rplC* and kanamycin resistance gene PCR products were used as templates for a PCR reaction with the kanamycin gene forward and the *rplC* reverse primer, resulting in a fused product between the two fragments (1198 kb in size). In the third step, equal amounts of the purified fusion product and the *rpsJ* fragment served as template and were fused into the 1500-bp full-length integration cassette in a PCR reaction with the *rpsJ* forward and the *rplC* reverse primer.

The resulting linear fragment, containing the kanamycin resistance gene flanked by the homologous sequences in the *rpsJ* operon, was transformed into *E. coli* BW 25113 containing the [pKD46] plasmid by electroporation. The *E. coli* BW 25113 [pKD46] electrocompetent cells were prepared from culture grown at 30°C in LB-medium with 0.2% arabinose (to induce the plasmid encoded recombination system) and 50 μg/ml ampicillin. After electroporation, cells were regenerated in 900 μl of LB medium additionally containing 0.2% arabinose and then incubated at 30°C for 2 h. Cells were plated on LB-agar plates containing 50 μg/ml kanamycin and incubated for 48 h at 30°C.

To verify that transformants growing on kanamycin-containing plates contained the correctly integrated kanamycin resistance gene, the entire integrated module was amplified from the strains by colony PCR and sequenced. Strains with correct integrations were subsequently cultivated at 37°C to induce loss of the recombination helper plasmid. The resulting strain was used as parent strain for the *rpsJ* mutants and was named *E. coli* BW KAN.

Initially, we intended to use a Cre recombinase encoding plasmid to excise the kanamycin cassette *via* its flanking loxP sites in the next step. As excision of the cassette was, however, lethal for unknown reasons (possibly due to problems caused by the short sequence remaining after excision), and our experiments showed that the strain containing the kanamycin cassette behaved similar to the wild-type strain, we continued our analyses with the cassette-containing strains.

#### Generation of *rpsJ* Mutants

The strain already carrying the integrated kanamycin resistance gene between the *rpsJ* and the *rplC* genes was used as template for PCRs to generate the integration cassettes for *rpsJ* mutant variants. The mutations were introduced in a two-step PCR procedure. In the first step, two overlapping fragments were generated by PCR using primers introducing the desired mutation. The purified overlapping fragments were then used as templates for a second PCR, in which the fragments were fused into the full-length linear integration cassettes that were subsequently transformed into *E. coli* and analyzed as described above.

To minimize the risk that the observed phenotypes are the consequence of additional mutations erroneously introduced during transformation, several clones per mutation were analyzed in our initial tests. After confirmation of similar behavior of the different clones, one clone each was chosen for the detailed study.

### Growth Curves

All growth experiments were inoculated from overnight cultures. These were inoculated with one colony and grown in 3 ml of Lysogenic Broth (LB) media. Growth curves were measured using a Bioscreen C MBR (Oy Growth Curves; Helsinki, Finland). The volume of culture was 200 μl of LB media with a start optical density at 600 nm of 0.05. Settings were continuous shaking at medium altitude, measurement every 10 min with a wide band filter (420–580 nm). Incubation temperatures were 20, 37, and 42°C. All strains were measured in three replicates during each experiment, and all experiments were performed three times. The mean values of the three replicates within the same experiment were used to calculate the total means and the standard deviation.

### Antibiotic Susceptibility Test

For determination of MICs, Etests according to EUCAST were performed for tigecycline, minocycline, tetracycline, ampicillin, chloramphenicol, erythromycin, and ciprofloxacin (EUCAST, 2020).

Broth microdilution in Mueller Hinton (MH) broth for tigecycline and tetracycline was performed according to EUCAST.

Tigecycline concentrations used for these experiments were as follows: 0.047, 0.065, 0.094, 0.125, 0.25, 0.38, 0.5, 0.75, 1.0, 1.5, and 2.0 μg/ml.

Tetracycline concentrations used for these experiments were as follows: 0.25, 0.38, 0.5, 0.75, 1.0, 1.5, 2.0, 2.5, 3.0, 3.5, and 4.0 μg/ml.

MICs were additionally determined for tetracycline and ampicillin in LB broth, with the same conditions as for the growth curves and with the addition of the antibiotics at different concentrations. For tetracycline, 37 and 42°C were used as incubation temperatures, while the curves of ampicillin-treated cultures were recorded at 37°C. MICs at 37°C incubation temperature were additionally determined for tetracycline in MH broth with the same conditions as for the growth curves and with the addition of tetracycline at different concentrations.

Tetracycline concentrations used for these experiments were as follows: 0.5, 0.75, 1.0, 1.5, 2.0, 2.5, 3.0, 4.0, and 5.0 μg/ml.

Ampicillin concentrations used for these experiments were as follows: 0.5, 1.0, 2.0, 3.0, 4.0, 6.0, 8.0, 10.0, and 12.0 μg/ml.

The MIC indicated in the results was the first concentration at which the optical density was less than 0.4 higher than the start OD after 24 and 48 h, respectively. All experiments were carried out three times. The median was determined from the individual MICs obtained.

### Stress Sensitivity Tests

The effect of abiotic stresses on S10 mutant strains was analyzed using spot assays. Overnight cultures grown in LB were diluted to an OD^600^ of one and then split into two aliquots (one for the heat-shock experiment, one for the other conditions). For the heat shock experiment, cells were then incubated at 50°C in a water bath for 10, 20, and 40 min. The heat shock was terminated by addition of an equal volume of room temperature (∼22°C) LB medium. Subsequently, cultures were diluted in 10-fold serial dilutions (10^–1^, 10^–2^, 10^–3^, and 10^–4^) in 96-well plates and then stamped onto LB-agar plates using a sterile metal stamp. The second aliquot was directly diluted by the addition of an equal volume of medium, followed by 10-fold serial dilutions and spotted onto plates containing mannitol (0.5, 0.75, or 1 M) or NaCl (0.5, 0.75, or 1 M) (stress conditions modified from Agrawal et al., 2015). All plates were incubated at 37°C for 8 h and photographed. For each of the stress conditions, the condition in which the greatest effects were observed/expected are shown, i.e., conditions in which the parent strain was still viable, but already showed a slight growth impairment. All spot tests were performed in triplicate.

## Results

### A Strategy to Genomically Integrate Mutant S10 Variants

Previous attempts have failed to chromosomally integrate *rpsJ* mutations in *E. coli* (Beabout et al., 2015). A likely explanation for difficulties with standard integration strategies is the fact that *rpsJ* is the first gene of a large operon (the *rpsJ* operon), and integration of *rpsJ* alleles together with a standard selection cassette, which carries its own promoter, might disturb normal expression of downstream genes in this operon. To avoid this, we generated a construct for integration of a kanamycin resistance gene without its own promoter as part of the *rpsJ* operon between the *rpsJ* and the *rpsC* gene (Figure 3, see “Materials and Methods” section for details). We then used this integration cassette to first generate a BW 25113 *E. coli* laboratory strain carrying the kanamycin resistance gene inserted 20 bp after the wild-type *rpsJ* gene (Figure 3).

**FIGURE 4:**
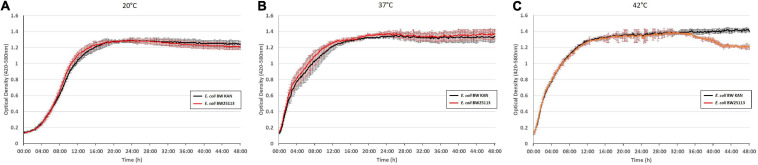
Integration of a kanamycin resistance gene into the *rpsJ* operon does not cause any significant growth defects. Growth curves of the *E. coli* BW 25113 laboratory strain (red) and the same strain carrying the kanamycin cassette in the *rpsJ* operon, *E. coli* BW KAN (black), at 20°C **(A)**, at 37°C **(B)**, and 42°C **(C)**.

To examine potential effects due to integration of the kanamycin resistance gene as an extra gene into the *rpsJ* operon, we compared the growth phenotype of BW 25113 with the same strain carrying the inserted kanamycin resistance gene (hereafter termed *E. coli* BW KAN) (Figure 4). There was no effect of the inserted kanamycin resistance gene on growth phenotypes at 20°C, 37°C, and 42°C during the first 36 h of growth. The only observed difference was the slight decrease in optical density at 42°C in *E. coli* BW 25113 carrying the kanamycin resistance gene after ∼36 h of incubation (Figure 4). Furthermore, no effect on susceptibility to the antibiotics tested in this study, i.e., tigecycline, tetracycline, minocycline, ampicillin, chloramphenicol, erythromycin, and ciprofloxacin (Tables 1–3), could be observed. We conclude that the insertion of the kanamycin resistance gene at the chosen position has negligible effects on growth and antibiotic resistance. Hence, this strategy is suitable to integrate *rpsJ* mutations into the chromosome.

**TABLE 1:** MICs determined by Etest.

Strains\Antibiotics	Tigecycline	Tetracycline	Minocycline	Ampicillin	Chloramphenicol	Erythromycin	Ciprofloxacin
	(μg/ml)	(μg/ml)	(μg/ml)	(μg/ml)	(μg/ml)	(μg/ml)	(μg/ml)
*E. coli* BW 25113 S10 WT	0.125	2.00	2.00	3.00	8.00	24.00	0.016
*E. coli* KAN S10 WT	0.125	2.00	2.00	3.00	8.00	24.00	0.016
*E. coli* KAN S10 ΔV57	0.38	3.00	4.00	3.00	8.00	16.00	0.016
*E. coli* KAN S10 V57G	0.19	3.00	3.00	3.00	8.00	24.00	0.016
*E. coli* KAN S10 V57A	0.125	2.00	1.50	3.00	8.00	16.00	0.016
*E. coli* KAN S10 V57L (CTC)	0.50	4.00	4.00	3.00	8.00	24.00	0.016
*E. coli* KAN S10 V57L (CTG)	0.50	4.00	4.00	3.00	8.00	24.00	0.016
*E. coli* KAN S10 V57I	0.38	3.00	3.00	3.00	8.00	16.00	0.016
*E. coli* KAN S10 V57D	0.38	3.00	3.00	3.00	8.00	24.00	0.016
*E. coli* KAN S10 V57N	0.19	3.00	3.00	3.00	8.00	16.00	0.016
*E. coli* KAN S10 V57K	0.094	0.75	1.50	3.00	8.00	16.00	0.016
*E. coli* KAN S10 V57R	0.19	3.00	2.00	3.00	8.00	24.00	0.016

**TABLE 2:** MICs determined by microdilution.

Strains\Antibiotics	Tigecycline (μg/ml)	Tetracycline (μg/ml)
*E. coli* BW 25113 S10 WT	0.094	0.50
*E. coli* KAN S10 WT	0.094	0.50
*E. coli* KAN S10 ΔV57	0.19	0.75
*E. coli* KAN S10 V57G	0.125	0.75
*E. coli* KAN S10 V57A	0.094	0.50
*E. coli* KAN S10 V57L (CTC)	0.25	1.00
*E. coli* KAN S10 V57L (CTG)	0.25	1.00
*E. coli* KAN S10 V57I	0.19	0.75
*E. coli* KAN S10 V57D	0.19	075
*E. coli* KAN S10 V57N	0.125	0.75
*E. coli* KAN S10 V57K	0.064	0.38
*E. coli* KAN S10 V57R	0.125	0.75

**TABLE 3:** MICs for tetracycline (37 and 42°C) and ampicillin (37°C incubation temperature) in liquid LB and MH media.

Antibiotic	Tetracycline						Ampicillin	
		
Temperature/Broth	37°C/MH		37°C/LB		42°C/LB		37°C/LB	
				
Time	24 h	48 h	24 h	48 h	24 h	48 h	24 h	48 h
Strains	(μg/ml)	(μg/ml)	(μg/ml)	(μg/ml)	(μg/ml)	(μg/ml)	(μg/ml)	(μg/ml)
*E. coli* BW 25113 S10 WT	0.75	1.00	1.00	1.50	1.00	1.50	6.00	8.00
*E. coli* KAN S10 WT	0.75	1.00	1.00	1.50	1.00	1.50	6.00	8.00
*E. coli* KAN S10 ΔV57	1.50	2.50	2.00	2.50	2.50	3.00	4.00	5.00
*E. coli* KAN S10V57G	1.50	1.50	2.00	2.00	2.50	2.50	6.00	8.00
*E. coli* KAN S10 V57A	0.75	1.00	1.00	1.50	1.00	1.50	6.00	8.00
*E. coli* KAN S10 V57L (CTC)	2.00	3.00	2.50	3.50	3.00	3.50	6.00	6.00
*E. coli* KAN S10 V57L (CTG)	2.00	3.00	2.50	3.50	3.00	3.50	6.00	6.00
*E. coli* KAN S10 V57I	1.00	1.50	1.50	2.00	2.00	2.00	6.00	8.00
*E. coli* KAN S10 V57D	1.50	1.50	1.50	2.00	2.00	2.50	6.00	8.00
*E. coli* KAN S10 V57N	1.00	1.50	1.50	2.00	2.00	2.50	6.00	8.00
*E. coli* KAN S10 V57K	0.50	0.75	0.75	1.00	0.75	1.00	6.00	8.00
*E. coli* KAN S10 V57R	1.00	1.50	1.50	2.00	1.50	2.00	6.00	8.00

### Mutations in the S10 Flexible Loop Result in Growth Defects

Next, using the same recombination strategy, we constructed strains carrying the following exchanges of valine 57 in the S10 flexible loop: V57L, V57I, and V57D, due to their appearance in tigecycline-resistant *E. coli* isolates; V57N to compare V57D to a similar amino acid exchange, but without introducing a negative charge; V57K to simulate the sequence commonly found in gram-positive bacteria and in *T. thermophilus* at this site; V57R to compare V57K to another mutation introducing a positive charge; and a V57 deletion, a V57G mutation, and a V57A mutation to evaluate the effects of complete deletion, size reduction, or just slight alteration of the S10 flexible loop tip residue. Moreover, in order to be able to investigate possible effects of non-optimal codon usage, two variants were created for the V57L exchange: one that corresponds to the DNA sequence in the observed isolates (S10V57L_CTC_) and a second that has the optimal codon usage for leucine (S10V57L_CTG_).

**FIGURE 5:**
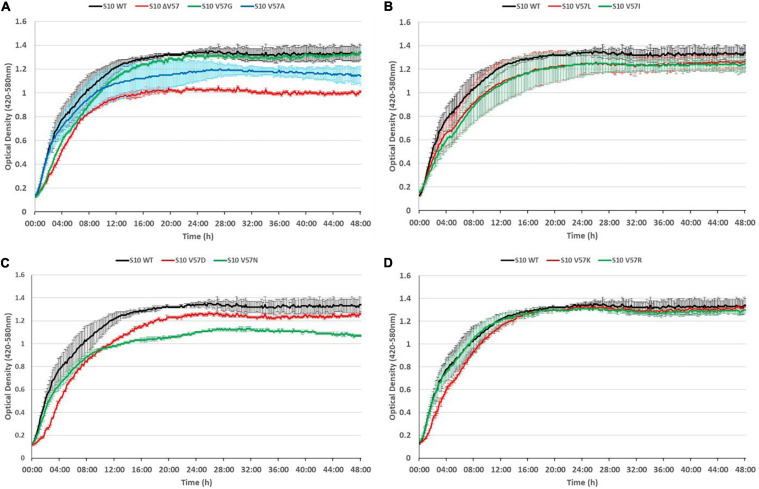
S10 mutations cause growth defects at 37°C. Growth curves of S10 mutants ΔV57, V57G, V57A **(A)**, V57L, V57I **(B)**, V57D, V57N **(C)**, V57K and V57R **(D)** compared to the control strain *E. coli* BW KAN (labeled S10 WT) at 37°C incubation temperature.

To evaluate potential phenotypic consequences of mutation of the S10 flexible loop, we recorded growth curves of all mutants compared to the corresponding S10 wild-type control strain *E. coli* BW KAN at the optimal growth temperature of 37°C. Defects compared to the control strain were, on the one hand, deduced from the reduced growth in logarithmic phase, as determined based on the optical density (OD) reached after 8 h of incubation and, on the other hand, from a lower maximum OD reached in the course of the cultivation (Figure 5).

As expected, the most severe growth defect, apparent from both a reduced OD after 8 h and a reduced maximum OD, was observed for the mutant in which V57 was deleted (ΔV57). The mutants carrying the known exchanges V57D, V57I, and V57L (irrespective of the codon) displayed mild growth defects in logarithmic phase (8 h) and also reached a lower maximum OD. Interestingly, the V57N mutant, resembling V57D without introducing a negative charge, grew faster than the V57D mutant in logarithmic phase, but reached a much lower maximum OD than V57D. Similarly, V57A displayed only mild defects in logarithmic phase but reached a lower maximum OD. Vice versa, the V57G and V57K mutants showed slower growth in the first 8 h of cultivation but reached the same maximum OD as the wild type. The only mutant displaying no growth defect in the tested conditions was V57R.

Summarizing, mutation of the S10 loop tip residue V57 leads in most instances to growth defects in *E. coli* already at optimal growth temperature, highlighting the functional importance of this amino acid.

**FIGURE 6:**
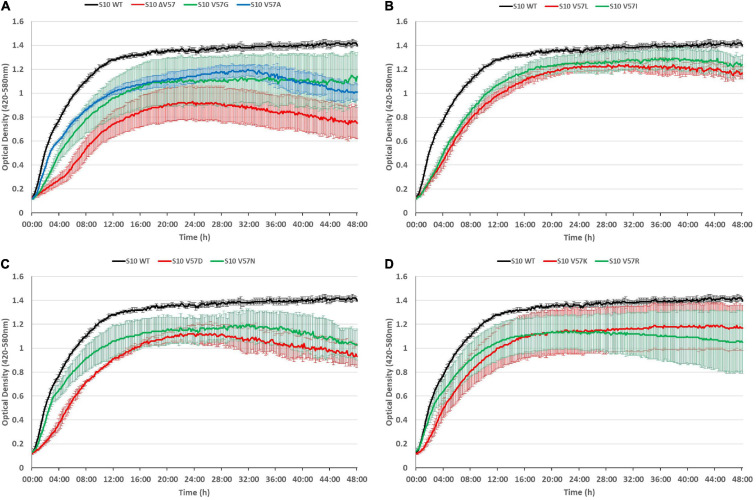
S10 mutations lead to thermosensitivity. Growth curves of S10 mutants ΔV57, V57G, V57A **(A)**, V57L, V57I **(B)**, V57D, V57N **(C)**, V57K and V57R **(D)** compared to the control strain *E. coli* BW KAN (labeled S10 WT) at 42°C incubation temperature.

It is frequently observed that phenotypes caused by point mutations are exacerbated at increased temperatures. To assess if this is also true for the S10 loop mutants, we also recorded growth curves at 42°C (Figure 6). At this condition, all mutants displayed growth defects compared to the S10 wild-type control. In most cases, incubation at 42°C further enhanced the growth defects compared to the 37°C incubation temperature. Even the mutants showing only mild defects at 37°C, i.e., V57R, V57G, and V57K, showed not only more severe defects at the 8-h time point, but also a reduced maximum OD at the 42°C incubation temperature. Similar enhancements of defects at 42°C compared to 37°C, and hence a thermosensitive phenotype, were also observed for the V57A, V57L, V57I, V57D, and ΔV57 mutant. The only mutant that showed no such thermosensitivity was V57N, for which the defect observed at 37°C was not further enhanced at 42°C. Hence, while V57N reached a lower OD than V57D at 37°C, it was the opposite at 42°C (Figures 5, 6).

To conclude, defects due to mutation of the S10 loop are in most cases further enhanced at 42°C.

**FIGURE 7:**
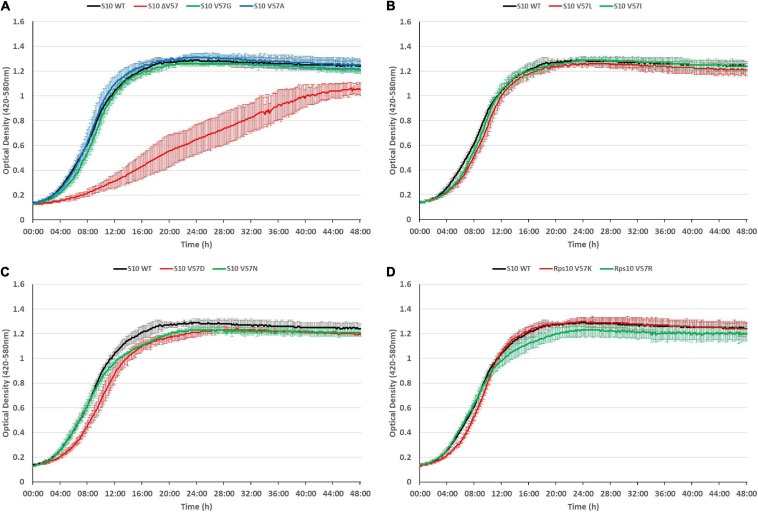
The S10 ΔV57 mutant is cold-sensitive at 20°C. Growth curves of S10 mutants ΔV57, V57G, V57A **(A)**, V57L, V57I **(B)**, V57D, V57N **(C)**, V57K and V57R **(D)** compared to the control strain *E. coli* BW KAN (labeled S10 WT) at 20°C incubation temperature.

The growth defects point toward a partial loss of function of S10, e.g., by affecting translation or ribosome biogenesis. Considering that ribosome biogenesis mutants frequently display cold-sensitive phenotypes (Dammel and Noller, 1995; Clatterbuck Soper et al., 2013; Stokes et al., 2014), we additionally tested for cold sensitivity by recording growth curves of the mutants at 20°C (Figure 7). However, most of the mutants even showed less defect at 20°C than at 37°C, with two exceptions. The V57R mutant showed reduced growth in the second 12 h of the growth curve and a reduced maximum OD at 20°C. The only mutant showing a strong defect at 20°C, similar to the phenotype at 42°C, was the ΔV57 mutant. Hence, the ΔV57 mutant is both thermo- and cryosensitive.

### Response of S10 Mutants to Abiotic Stresses

**FIGURE 8:**
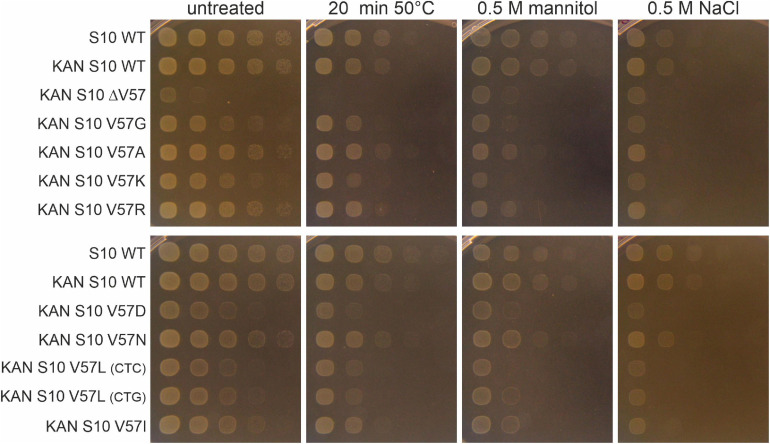
Spot assays of S10 mutants under different abiotic stresses. Cells were either exposed to heat shock (20 min at 50°C), diluted in 10-fold serial dilutions and then spotted on LB-agar plates, or diluted and spotted on LB-plates containing mannitol or NaCl. Pictures were taken after 8 h of incubation at 37°C.

The growth differences at different temperatures (in particular the thermosensitivity of most mutants) point to a partial infunctionality of the S10 protein in the mutants. This frequently also results in increased stress sensitivity. To study that, we exposed the cells to heat (50°C), osmotic (mannitol), and salt (NaCl) stress (Figure 8). Notably, heat shock at 50°C for 20 min was lethal for the ΔV57 mutant, while all other mutants were affected by heat shock to a similar extent as wild type. The opposite was true for mannitol and NaCl—while the ΔV57 mutant grew normally under these conditions, most other mutants appeared to be slightly more affected than the wild type. Only the V57N mutant was largely unaffected by the stresses. Additionally, a slight difference in growth on mannitol containing media was observed for the variants carrying the two V57L mutants with the different codons. The variant with the more frequently used codon, CTG, grew slightly better on mannitol than the variant with the less used codon CTC, suggesting that the presumably lower protein level in the mutant with the rare codon might slightly increase the sensitivity to mannitol. To summarize, the only mutant showing a strong defect upon exposure to stress was the ΔV57 mutant, which displayed a reduced heat shock tolerance.

### S10 Flexible Loop Mutations Alter the Susceptibility to Tetracycline, Minocycline, and Tigecycline

Next, we aimed at determining the effects of mutation of S10 V57 on tigecycline, minocycline, and tetracycline resistance. Moreover, to be able to evaluate the specificity of potential changes in resistance, we also determined susceptibility to antibiotics unrelated to tetracyclines, i.e., ampicillin, chloramphenicol, ciprofloxacin, and erythromycin. MICs of all these antibiotics were determined by Etest. Furthermore, MICs for tigecycline and tetracycline were also confirmed by broth microdilution in MH.

MICs to tetracycline and ampicillin were additionally determined based on OD measurement and recording of growth curves in liquid media (MH and LB), at 37 and 42°C.

Susceptibility to neither ampicillin, nor chloramphenicol, nor ciprofloxacin was changed in any of the mutants as determined by Etest (Table 1). Also, the erythromycin Etest result was in a similar range for all strains; however, the fact that the inhibiting areola ellipse was for five strains slightly above and seven strains lightly below the 16 μg/ml mark resulted in the different readouts of the MICs as 24 and 16 μg/ml, respectively.

In contrast to these control antibiotics, all but one S10 mutations changed the MICs for tetracycline, minocycline, and tigecycline (Tables 1–3).

The V57L mutants displayed the highest MICs for tetracycline (4.00 μg/ml), minocycline (4.00 μg/ml), and tigecycline (0.50 μg/ml) in Etest, in line with the occurrence of this exchange in tigecycline-resistant isolates. In addition, the two other mutants previously observed in resistant isolates, i.e., V57I and V57D, as well as the V57 deletion mutant, showed increased MICs. In contrast, the V57G, V57N, and V57R mutant displayed only a minimal, although reproducible increase in MICs. No changes in the MICs were identified for the V57A mutant.

In contrast, the V57K mutant displayed a very unexpected phenotype, showing a decreased MIC and hence increased sensitivity to tetracycline (0.75 μg/ml), and also slightly decreased MICs to minocycline (1.50 μg/ml) and tigecycline (0.094 μg/ml) in Etest (Table 1).

MIC determination by broth microdilution confirmed the effects of all mutants, compared to the parent strain, on tigecycline and tetracycline resistance, even though the MIC levels for both antibiotics were generally lower (Table 2).

Last but not least, MICs also determined for tetracycline in LB and MH broth liquid culture confirmed the trends observed in Etest and broth microdilution (Table 3).

**FIGURE 9:**
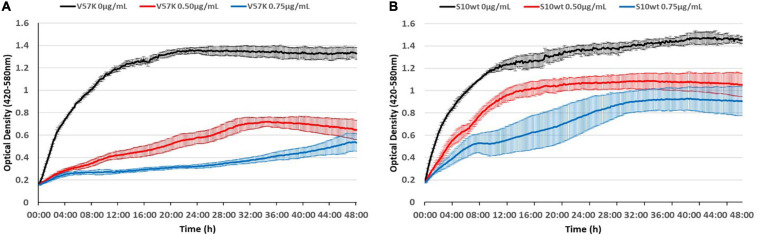
The S10 V57K mutant is hypersensitive to tetracycline. Influence of different tetracycline concentrations (0 μg/ml black, 0.5 μg/ml red, and 0.75 μg/ml blue) on *E. coli* KAN S10 V57K **(A)** and *E. coli* KAN S10 WT **(B)**.

As the reduced MIC of the V57K mutant was surprising, we wanted to visualize this effect better throughout growth. To this end, we recorded growth curves in the presence of different tetracycline concentrations, further demonstrating the hypersensitivity of this strain to tetracycline (Figure 9).

Last but not least, we evaluated the possibility that the mechanism responsible for the thermosensitive phenotype of most of the tested mutants might also lead to higher MICs at 42°C. However, the influence of higher temperature on the MIC to tetracycline was only minimal (Table 3).

We conclude that the identity of the amino acid at position 57 of *E. coli* ribosomal protein S10 is an important determinant for the susceptibility to tetracyclines including tigecycline.

Considering the much higher tigecycline/tetracycline resistance observed for *E. coli* S10 loop mutants observed in previous studies, the MICs we determined with our mutants were surprisingly low. To make a direct comparison, we determined the MICs of a V57L and a V57D mutant generated in the Shamoo group by selection on tigecycline and compared them to our mutants carrying the same amino acid exchanges (Table 4; Beabout et al., 2015).

**TABLE 4:** MICs determined by Etest.

Strains\Antibiotics	Tigecycline (μg/ml)	Tetracycline (μg/ml)
*E. coli* BW 25113 S10 WT	0.125	2.00
*E. coli* KAN S10 WT	0.125	2.00
*E. coli* KAN S10 V57L (CTC)	0.50	4.00
*E. coli* KAN S10 V57L (CTG)	0.50	4.00
*E. coli* KAN S10 V57D	0.38	3.00
*E. coli* BW P3 S10 V57L (CTC)	12.00	48.00
*E. coli* BW P6 S10 V57D	6.00	64.00

Strikingly, the mutants selected under tigecycline pressure showed much higher MICs, reaching MICs to tigecycline of 12.0 μg/ml (V57L) or 6.0 μg/ml (V57D), and MICs to tetracycline of 48 (V57L) or 64 (V57D). We speculate that, in these mutants, the selection pressure led to the appearance of other resistance-causing mutations in addition to the mutations in S10.

## Discussion

Our data reveal for the first time that mutation of a single residue, V57, in the S10 flexible loop, is sufficient to cause altered tetracycline, minocycline, and tigecycline susceptibility in *E. coli*.

The highest increase of MICs to tetracycline, minocycline, and tigecycline was observed in the V57L mutant, with a fourfold increased MIC to tigecycline and a twofold increased MIC to tetracycline according to Etest data. Nevertheless, the tigecycline-MIC of 0.5 μg/ml observed in Etest for the V57L mutant is still too low to be classified tigecycline resistance according to EUCAST (which would require a MIC higher than 0.5 μg/ml). Therefore, we presume that the reported cases of high-level resistance of Enterobacteriaceae with S10 V57D, V57I, or V57L mutations are very likely not caused by S10 mutation alone, but by combination of the S10 mutation with additional mutations, for example in ribosomal RNA or proteins (Villa et al., 2014; Beabout et al., 2015). This assumption is also confirmed by the comparative data with the V57D and V57L isolates provided by the Shamoo group, which revealed that the selected mutants showed much higher resistance to tigecycline and tetracycline than our mutants. We observed that these mutants also showed resistance to other antibiotics (ampicillin and chloramphenicol), which is a hint that these isolates may have collected also mutations affecting general resistance mechanisms, e.g., *via* efflux pumps (data not shown).

Also, several other novel (V57N, V57G, and V57R) exchanges and even the deletion of V57 resulted in increased MICs to tetracyclines and tigecycline. However, except for the V57 deletion mutant, the increase in MIC was very small, making it difficult to make definite conclusions based on the current data. Therefore, it is difficult to speak of an efficacious effect on tigecycline susceptibility in the case of the V57G, V57N, and V57R mutants.

In contrast, the replacement of V57 by K, the residue present at this position in many gram-positive bacteria like Staphylococci, Streptococci, and Enterococci, but also in the gram-negative *T. thermophilus*, surprisingly resulted in a decreased MIC, and hence a higher sensitivity to tetracycline, minocycline, and tigecycline (Meagher et al., 2005; Jenner et al., 2013). The effect on tetracycline was significantly higher than on tigecycline and the structurally related minocycline. Cocozaki et al. (2016) also reported mutations on the ribosome (16S rRNA) that increase tetracycline sensitivity but not tigecycline sensitivity. This could be due to the fact that the already very good binding activity of tigecycline can only be improved to a small extent or with difficulty by mutations at or nearby the target site.

Notably, S10 V57 is not in direct contact with tigecycline. However, both the S10 flexible loop and tigecycline are in contact with 16S rRNA helix h31; therefore, we speculate that mutations in V57 alter the positioning of h31, which in turn might affect tigecycline binding and/or action. Reducing the size of the loop tip residue (amino acid 57) or completely deleting it might abolish S10 interaction with the loop and therefore increase its flexibility, whereas larger or negatively charged amino acids may even lead to repulsion of h31. We observed that not only the reduction but also the increase in size of residue 57 increases the MIC to tigecycline; hence, we speculate that in both cases, h31 becomes mispositioned, which may reduce tigecycline affinity to this site or even abolish the interaction of tigecycline with helix h31. In that case, only the interactions of tigecycline with helix h34 would be retained, which would presumably lead to a reduced overall affinity of tigecycline to the 30S subunit. Interestingly, the exchange of valine 57 to lysine was the only one that increased sensitivity to tetracycline, and to some extent also to tigecycline and minocycline. We hypothesize that the exchange to lysine may strengthen the interaction of S10 with h31, potentially through its positively charged side chain, which may engage in an ionic interaction with the phosphate backbone of the h31 rRNA. This may fix h31 in a more stable position, which, in turn, may facilitate tigecycline binding and/or action (Jenner et al., 2013; Cocozaki et al., 2016). Interestingly, the loop tip residue in gram-positive bacteria and *T. thermophilus* is naturally a lysine. Remarkably, despite the different loop sequences, the S10 loop conformation of *T. thermophilus* and *E. coli* is highly similar, and in both cases, the loop tip residue is oriented toward the 2′OH group of A964 (only two nucleotides apart from the tigecycline binding site at G966) (Jenner et al., 2013; Cocozaki et al., 2016). It is therefore tempting to speculate that the V57K exchange brings the lysine into the correct position for the ionic interaction with A964, just like the lysine at the same position in *T. thermophilus* S10.

Surprisingly, the V57R mutant, also introducing a positive charge, did not result in decreased sensitivity to tetracyclines. We speculate that not only the positive charge but also the size of the residue is relevant. The positive charge introduced by the V57R exchange is likely in a different position than the positive charge in the V57K mutant and hence not able to undergo the same interactions. In the future, structural studies would be helpful to better understand the mechanisms by which the different exchanges alter tetracycline/tigecycline sensitivity.

Still, the question remains why some bacteria carry a valine and others a lysine as the S10 loop tip residue. We speculate that due to slight differences in the ribosome structure, a valine is more favorable in gram-negative and a lysine more favorable in gram-positive bacteria for full functionality of S10. Indeed, S10 V57K was not fully functional in *E. coli*, as concluded from the growth defects of this mutant.

Also, most other mutations introduced in this study—except for the V57R mutation—resulted in growth defects at 37°C, which were in most cases further enhanced at 42°C. Moreover, the ΔV57 mutant was cold-sensitive and sensitive to heat shock. The severity of growth defects was not in correlation with the extent of tigecycline resistance. For example, while the V57L and the ΔV57 mutant both showed an increased MIC to tested tetracyclines including tigecycline, V57L resulted in only a mild growth defect and ΔV57 resulted in a severe growth defect. Hence, beside the role of V57 in affecting tigecycline effectiveness, V57 is also important for the full functionality of ribosomal protein S10.

In addition to its roles in ribosome biogenesis and translation, S10 has an extra-ribosomal function in transcription antitermination. We can, however, exclude that our mutants are impaired in antitermination, as it has been shown that the S10 flexible loop is dispensable for the transcriptional function of S10, and even an S10 variant deleted for the entire loop is fully functional in antitermination (Luo et al., 2008).

We consider it more likely that the function of S10 in translation or ribosome biogenesis is affected in the mutants. Interestingly, the flexible loop of the *Saccharomyces cerevisiae* S10 ortholog (uS10/Rps20) was recently shown to be important for the coordination of late small ribosomal subunit maturation steps (Mitterer et al., 2019). Despite great differences in the ribosome biogenesis pathways between prokaryotes and eukaryotes, core elements of the pathway, like the assembly of ribosomal proteins, are conserved. Beside the known connection of ribosome biogenesis mutants with cold sensitivity, thermosensitivity has also been found in some ribosome biogenesis mutants, and the late stages of ribosomal subunit biogenesis (in which S10 is incorporated) have been described to be sensitive to heat stress (Rene and Alix, 2011; Shajani et al., 2011). Whether the growth defects and thermosensitivity of our mutants, as well as the cold sensitivity and heat shock sensitivity of the ΔV57 mutant, are indeed due to ribosome biogenesis defects will be the subject of future studies.

In summary, our study has demonstrated that the identity of the amino acid at position 57 in *E. coli* ribosomal protein S10 is critical both for the function of the ribosomal protein and for tigecycline susceptibility. Our newly generated mutants can provide a basis for future biochemical and structural studies to unravel the exact mechanisms by which different mutations in S10 lead to altered susceptibility to tetracyclines and tigecycline.

## Data Availability Statement

The raw data supporting the conclusions of this article will be made available by the authors, without undue reservation.

## Author Contributions

GZ, BP, and GK designed the project and the experimental setups. NI, CW, BP, MC, and GZ performed the experiments. NI, CW, CK, BP, MC, and GZ performed the data analysis. GZ and BP wrote the manuscript. All authors commented on and approved the manuscript.

## Conflict of Interest

The authors declare that the research was conducted in the absence of any commercial or financial relationships that could be construed as a potential conflict of interest.
