# Supplementary material for: Effects of Ribosomal Protein S10 Flexible Loop Mutations on Tetracycline and Tigecycline Susceptibility of Escherichia coli
Source: Front Microbiol. 2021 Jun 18;12:663835. doi: 10.3389/fmicb.2021.663835 (PMC8249722; doi:10.3389/fmicb.2021.663835)
Supplement: Supplementary file 1 [file Table_1.DOCX]

| Primer | Sequence 5’-3’ | Comments |
| --- | --- | --- |
| rpsJtotal-FW | GAAAATGAATTCAAAATACTGTGGAAG | binds 105 to 78 bp before the *rpsJ* gene*.* For sequencing of total *rpsJ* |
| rpsJ-Fragment-Fw | AAAGCGTTTGATCATCGTC | Forward primer for amplification of *rpsJ* PCR fragment; binds to bases 31 to 50 of *rpsJ* |
| rpsJ-Fragment-Rv | GTATGCTATACGAAGTTATTCAATCGCTCAATGACCTG | Reverse primer for *rpsJ* fragment amplification. The black part indicates the sequence homologous to base 304 to 314 (counted from the start of the *rpsJ* gene); the red part indicates the introduced overlap containing the first 19 bases of the kanamycin resistance cassette (comprising a loxP site) |
| KanLoxP-Fw | GAGCGATTGAATAACTTCGTATAGCATACATTATACGAAGTTATATGCAAGGGGTGTTATGAGCC | Forward primer for Kan-Fragment: black: the *rpsJ* operon sequence from position 304 to 314 (from the start of the *rpsJ* gene), red: loxP site, blue: the first 21 nucleotides of the kanamycin gene |
| KanLoxP-Rv | GTATGCTATACGAAGTTATCTTAGAAAAACTCATCGAGCATC | Reverse primer for Kan-Fragment: red: 19 bases comprising a loxP site directly after the kanamycin resistance gene; blue: last 24 nucleotides of kanamycin resistance gene (blue) |
| rplCFragment Fw | TTTTTCTAAGATAACTTCGTATAGCATACATTATACGAAGTTATGAGGTTGAAACAATGATTGG | Forward primer for *rpsC* PCR-fragment. Blue: Last nine nucleotides of kanamycin resistance gene; Red: loXP site; Black: last 10 nt of the kanamycin resistance gene |
| rplCFragment-Rv | AATGCTCTGACCTACAGTG | Reverse primer for *rpsC* PCR fragment binding to nts 271-289 of the coding sequence of *rplC* |
| S10Delta57-Fw | TCTCCCCGCACAACAAAGACGCGCGCGATCAG | Forward primer excluding nt 169-171 (encoding V57) of the *rpsJ* gene; used for generation of the delta V57 mutant |
| S10Delta57-Rv | GTCTTTGTTGTGCGGGGAGATCAGAACAGTG | Reverse primer excluding nt 169-171 (encoding V57) of the *rpsJ* gene; used for generation of the delta V57 mutant |
| S10V57D-Fw | CTGATCTCCCCGCACGACAACAAAGACGCGCGC | Forward primer for exchange of nucleotides at position 169-171 (red) in order to code for Aspartic acid (D) |
| S10V57D-Rv | GCGCGCGTCTTTGTTGTCGTGCGGGGAGATCAG | Reverse primer for exchange of nucleotides at position 169-171 (red) in order to code for Aspartic acid (D) |
| S10V57G-Fw | CTGATCTCCCCGCACGGCAACAAAGACGCGCGC | Forward primer for exchange of nucleotides at position 169-171 (red) in order to code for Glycine (G) |
| S10V57G-Rv | GCGCGCGTCTTTGTTGCCGTGCGGGGAGATCAG | Reverse primer for exchange of nucleotides at position 169-171 (red) in order to code for Glycine (G) |
| S10V57K-Fw | CTGATCTCCCCGCACAAGAACAAAGACGCGCGC | Forward primer for exchange of nucleotides at position 169-171 (red) in order to code for Lysine (K) |
| S10V57K-Rv | GCGCGCGTCTTTGTTCTTGTGCGGGGAGATCAG | Reverse primer for exchange of nucleotides at position 169-171 (red) in order to code for Lysine (K) |
| S10V57A-Fw | CTGATCTCCCCGCACGCGAACAAAGACGCGCGC | Forward primer for exchange of nucleotides at position 169-171 (red) in order to code for Alanine (A) |
| S10V57A-Rv | GCGCGCGTCTTTGTTCGCGTGCGGGGAGATCAG | Reverse primer for exchange of nucleotides at position 169-171 (red) in order to code for Alanine (A) |
| S10V57R-Fw | CTGATCTCCCCGCACCGCAACAAAGACGCGCGC | Forward primer for exchange of nucleotides at position 169-171 (red) in order to code for Arginine (R) |
| S10V57R-Rv | GCGCGCGTCTTTGTTGCGGTGCGGGGAGATCAG | Reverse primer for exchange of nucleotides at position 169-171 (red) in order to code for Arginine (R) |
| S10V57N-Fw | CTGATCTCCCCGCACAACAACAAAGACGCGCGC | Forward primer for exchange of nucleotides at position 169-171 (red) in order to code for Asparagine (N) |
| S10V57N-Rv | GCGCGCGTCTTTGTTTTGGTGCGGGGAGATCAG | Reverse primer for exchange of nucleotides at position 169-171 (red) in order to code for Asparagine (N) |
